# Mesenchymal stem cells for osteoarthritis: Recent advances in related cell therapy

**DOI:** 10.1002/btm2.10701

**Published:** 2024-08-05

**Authors:** Jianjing Lin, Jingtao Huang, Zilu Jiao, Mengyuan Nian, Canfeng Li, Yali Dai, Shicheng Jia, Xintao Zhang

**Affiliations:** ^1^ Department of Sports Medicine and Rehabilitation Peking University Shenzhen Hospital Shenzhen China; ^2^ Shantou University Medical College Shantou China; ^3^ Cardre Health Care Department Peking University Shenzhen Hospital Shenzhen China

**Keywords:** cartilage, extracellular vesicles, mesenchymal stem cells, osteoarthritis, stem cell

## Abstract

Osteoarthritis (OA) is a degenerative joint disease that affects the entire joint and has been a huge burden on the health care system worldwide. Although traditional therapy and targeted cartilage cell therapy have made significant progress in the treatment of OA and cartilage regeneration, there are still many problems. Mesenchymal stem cells from various tissues are the most studied cell type and have been used in preclinical and clinical studies of OA, because they are more widely available, have a greater capacity for in *vitro* expansion, and have anti‐inflammatory and immunomodulatory properties compared to autologous chondrocytes. This article will systematically review the latest developments in these areas. It may provide new insights for improving OA and cartilage regeneration.

**Abbreviations**
ACL: anterior cruciate ligament
ACLT: anterior cruciate ligament transection
ADSCs: adipose‐derived mesenchymal stem cells
BMAC: bone marrow aspiration and concentration
BMMSCs: bone marrow mesenchymal stem cells
CFU: colony‐forming units
CIOA: collagenase‐induced osteoarthritis
CRISPR: clustered Regularly Interspersed Short Palindromic Repeats
Col II/COL2: collagen type II
COX2: cyclooxygenase 2
CT: computed tomography
DMEM: Dulbecco's modified eagle medium
DPC: dipropyl carbonate
ESCs: embryonic stem cells
EVs: extracellular vesicles
FCD: full‐thickness cartilage defects
GAG: glycosaminoglycan
HO‐1: heme oxygenase‐1
hBMSC: human bone marrow‐derived stem cells
hSMSC: human sinus membrane‐derived stem cells
IFP‐MSCs: infrapatellar fat pad‐derived MSCs
IGF‐1: insulin growth factor‐1
IL‐1Ra: interleukin‐1 receptor antagonist
IL‐1β: interleukin 1β
IL‐4: interleukin‐4
iPSCs: induced pluripotent stem cells
LIPUS: low‐intensity pulsed ultrasound
LPS: lipopolysaccharide
MCH: microheterogenic collagen‐based hydrogel
MCL: medial collateral ligament
MIA: monoiodoacetate
MMT: medial meniscal tear
MR: magnetic resonance
MRI: magnetic resonance imaging
MSC‐GFP: MSC‐Green fluorescent protein
MSCs: mesenchymal stem cells
NMSCs: neonatal mesenchymal stem cells
NSAIDs: non‐steroidal anti‐inflammatory drugs
nsPEFs: nanosecond pulsed electric fields
OA: osteoarthritis
PAM: polyacrylamide
PCD: partial‐thickness cartilage defects
PDGF: platelet‐derived growth factor
PMSCs: placental mesenchymal stem cells
PPG: palmitic acid protein G
PRP: platelet‐rich plasma
PSC‐MSCs: pluripotent stem cell‐derived MSCs
PWL: paw‐withdrawal latency
PWT: paw‐withdrawal threshold
qRT‐PCR: quantitative reverse transcription‐polymerase chain reaction
qPCR: quantitative polymerase chain reaction
SD: standard deviation
SDSCs: synovium‐derived stem cells
SMMSCs: synovial membrane‐derived mesenchymal stem cells
SPIO: superparamagnetic iron oxide
TGase2: transglutaminase 2
TGF‐β3: transforming growth factor‐β3
TGFBI: TGF‐β‐induced
tMSCs: TP‐modified MSCs
TNF‐α: tumor necrosis factor α
UCB‐MSCs: umbilical cord blood‐derived mesenchymal stem cells
UC‐MSCs: umbilical cord‐derived mesenchymal stem cells

**Translational Impact Statement**

This review highlights the potential of mesenchymal stem cells (MSCs) from various tissues as a promising treatment for osteoarthritis (OA) and cartilage regeneration, due to there is currently no optimal clinical therapy for OA and MSCs' availability, expansion capacity, and therapeutic properties including anti‐inflammatory and immunomodulatory effects. Addressing the growing global burden of OA, MSC therapies could significantly enhance quality of life and reduce healthcare costs by improving treatment outcomes and possibly reversing damage rather than merely alleviating symptoms.

## 1 INTRODUCTION

Osteoarthritis (OA) is a common chronic joint disease that affects approximately 500 million people worldwide, accounting for 7% of the global population.^1^ Demographic changes are driving the increased prevalence of OA and thus the burden of OA is increasing faster than other diseases.^2^ From 1990 to 2019, the number of people affected by OA increased by 48% globally, and OA is already the 15th leading cause of disability worldwide, even is expected to become the 4th leading cause of disability worldwide by 2020.^1^, ^3^ Take China for instance, as one of the countries with the highest burden of OA, the prevalence of symptomatic knee OA in middle‐aged and elderly people aged 45 years or older is as high as 8.1% (10.3% in women and 5.7% in men), and even up to 13.7% in certain regions. It is estimated that more than 100 million people suffer from OA in China.^4^ With an aging population, an increasing prevalence of obesity and joint damage, the number of people affected by OA will increase in the future. The number of people affected by OA will increase by approximately 50% in the next 20 years.^2^ OA not only has a serious impact on the quality of life of individuals but also places a huge burden on healthcare systems and is now a major challenge for researchers and joint physicians worldwide.

OA is a total joint disease that includes cartilage destruction, subchondral bone remodeling and sclerosis, ligament dysfunction, synovial hypertrophy, osteophyte formation, meniscal damage, and periarticular muscle atrophy,^5^ with the destruction of articular cartilage being the hallmark pathological change in OA. The current treatment modalities for OA include non‐pharmacological interventions, pharmacological treatment, and surgical procedures. Non‐pharmacological interventions suggest self‐management, regular exercise, and weight control for OA patients and are considered the first line of OA treatment.^6^ However, adherence to exercise in patients with OA decreases over time, and there are no recommended exercise intensities with precise guidelines due to the lack of quantitative studies on exercise intensity and cartilage damage repair. Exercise therapy is also not commonly recommended by physicians for safety reasons. Drug therapy consists of systemic administration and is usually divided into oral non‐steroidal anti‐inflammatory drugs (NSAIDs) or selective cyclooxygenase 2 (COX2) inhibitors. But they increase the risk of gastrointestinal bleeding and cardiovascular disease,^7^ Osteoarthritis Research Society International recommends that Intra‐articular hyaluronic acid (IAHA) is endorsed for longer‐term therapeutic efficacy, given its correlation with symptom amelioration lasting beyond 12 weeks and manifestation of a favorable safety profile.^8^ The risk of infection in surgical treatments such as arthroplasty, arthroscopic surgery, and osteotomy cannot be ignored, and studies have found that quadriceps strength, voluntary muscle activation, and function are significantly impaired in patients after total knee arthroplasty and that strength and function do not return to normal levels in the same age group after several years.^9^ Besides, joint revisions are performed in 6% and 12% of cases within 5 and 10 years, respectively.^10^ In addition, the conventional treatments described above aim to reduce pain and improve joint mobility in OA patients, but do not promote the regeneration of damaged articular cartilage, and it is important to find new effective treatments to deal with OA.

**FIGURE 1:**
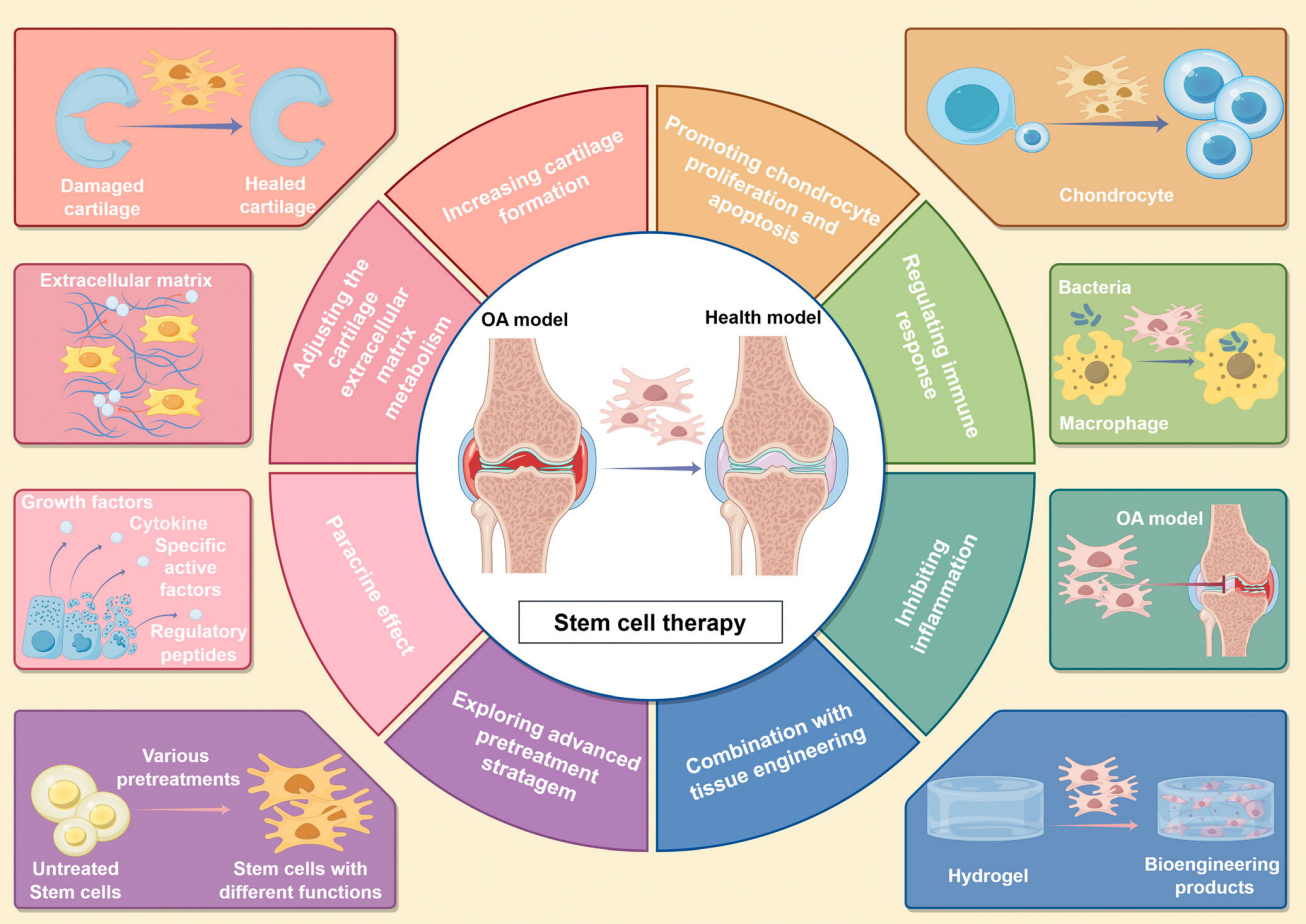
Role of stem cell therapy in the treatment of OA. Created with figdraw.com. OA, Osteoarthritis.

Cell therapy is considered a promising approach for the treatment of OA and cartilage regeneration. In recent years, autologous chondrocyte or stem cell‐based approaches have emerged as promising options for OA treatment.^11^ Although autologous chondrocytes are safe and efficient, their limited availability and ability to dedifferentiate and their loss of function during in *vitro* expansion limit their use in OA therapy.^12^ Compared to autologous chondrocytes, stem cells are more widely available, have a greater capacity for in *vitro* expansion, and have anti‐inflammatory and immunomodulatory properties.^13^ Mesenchymal stem/stromal cells (MSCs) from various tissues are the most studied cell types and have been used in preclinical and clinical studies of OA.^14^, ^15^ As stem cell therapy is considered to demonstrate its therapeutic effects in a paracrine way,^16^ stem cell‐derived extracellular vesicles (EVs), especially exosomes, are emerging as a novel and effective alternative to stem cells as cell‐free therapies for the treatment of many diseases, including OA.^17^ In this review, we first introduce the role of stem cell therapy in the treatment of OA (Figure 1). Then, we review the application of stem cells of varied origins and their use in the treatment of OA. Finally, we summarize the research progress of tissue engineering in stem cell research related to OA.

## 2 TREATMENT OF OA WITH STEM CELLS FROM DIFFERENT SOURCES

In recent years, more and more studies have shown that different types of stem cells have therapeutic effects on OA and cartilage injury. MSCs are currently the most studied cell type. In the past few years, research on pluripotent stem cells has also been rapidly developed, in which both Embryonic stem cells (ESCs) and induced pluripotent stem cells (iPSCs) can be used as a source of infinitely differentiable cells in cell therapy.^18^

### 2.1 Mesenchymal stem cell

MSCs are multipotent progenitor cells originally found in bone marrow, but can also be found in many other tissues, including adipose tissue, dental pulp, synovial membrane, placenta, umbilical cord, and cord blood.^19^ They can proliferate in *vitro* and can differentiate into several different cell types, such as adipocytes, chondrocytes and osteoblasts. Wyles et al. concluded that there is ample evidence to support MSCs guiding regeneration through paracrine stimulation of natural tissue.^20^ Bone marrow and adipose tissue have been the most used sources of MSCs because of the simplicity of the derivation technique and their large number, based on these, they have been studied extensively in OA therapy.

**FIGURE 2:**
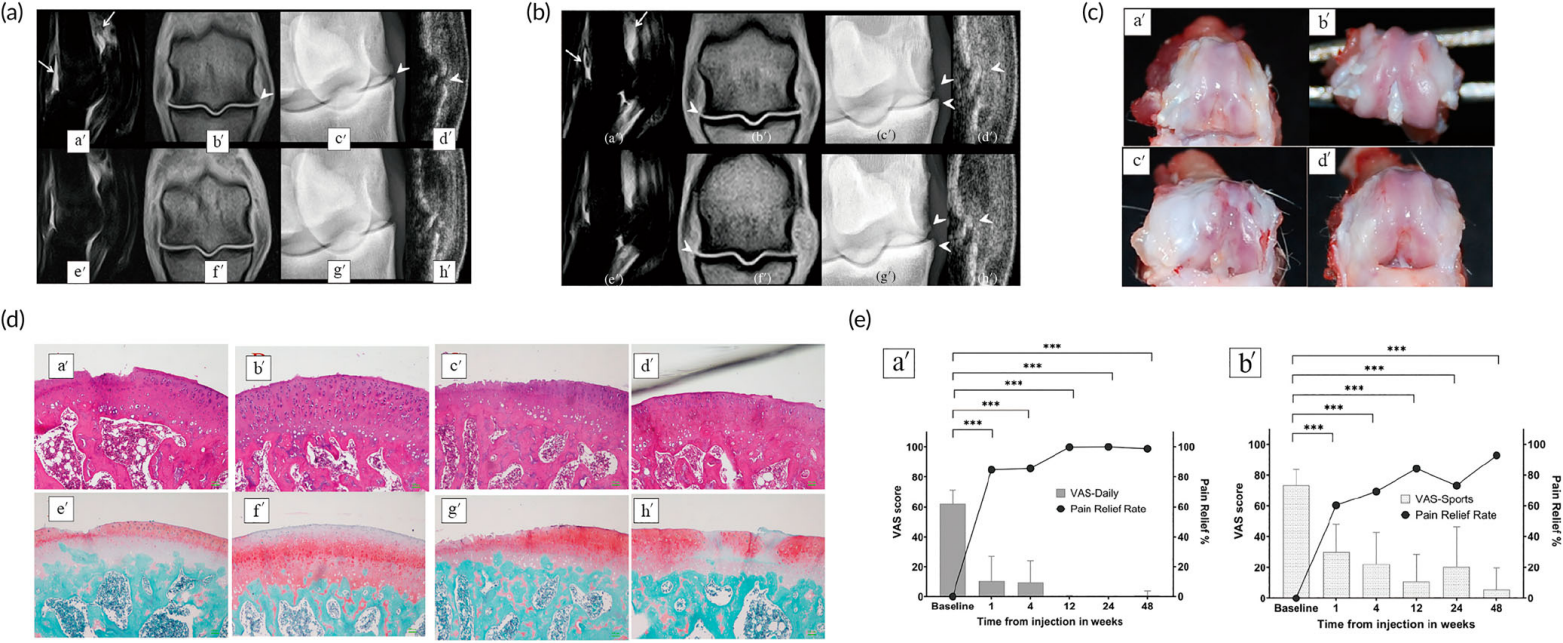
Therapeutic effects of different MSCs on cartilage defect. (a) Week 12 Comparative Imaging of Horse 7's Metacarpophalangeal Joints: The left fore fetlock (images a′–d′) treated with placebo shows grade 3 synovial effusion and periarticular osteophytes on STIR sagittal and T1‐weighted dorsal MRI images, respectively (white arrows, arrowheads in a′, b′). In contrast, the right fore fetlock (images e′–h′) treated with BMMSCs exhibits grade 2 synovial effusion and lower osteophyte scores (grade 3 on the left vs. grade 1 on the right in 35° oblique radiographs and ultrasound). (b) Week 12 Comparative Imaging of Horse 4's Metacarpophalangeal Joints: The left fore fetlock (images a′–d′) receiving placebo shows grade 3 synovial effusion and periarticular osteophytes on STIR sagittal and T1‐weighted dorsal MRI images (white arrows, arrowheads in a′, b′). The right fore fetlock (images e′–h′) treated with UCB‐MSCs displays grade 2 synovial effusion. Osteophytes are consistent at grade 2 in 35° oblique radiographs and ultrasounds for both fetlocks (arrow heads in c′, g′, d′, h′). Adapted with permission.^29^ Copyright © 2021 The Author(s). (b) Gross morphological observations of femoral condyles were conducted at weeks 8 and 12 post‐surgery. Groups included control and ADSC‐treated at both time points. Severe erosion was observed in the control group at week 12, while moderate erosion was noted in both control and ADSC‐treated groups at week 8 and 12. Mild lesions were seen in the ADSC‐treated group at week 8. (c) Histological evaluation of femoral condyles was conducted at weeks 8 and 12 post‐surgery. Specimens were stained with hematoxylin and eosin (H&E) and Safranin‐O/fast green. Severe cartilage damage was observed in groups c′ and g′, moderate damage in groups a′, e′, d′, and h′, and mild damage in groups b′ and f′. Adapted with permission.^43^ Copyright © 2017 The Author(s). (d) Changes in visual analog scale (VAS) scores for daily (a′) and sports (b′) activities were monitored over 48 weeks post infrapatellar fat pad‐derived MSCs (IFP‐MSCs) injection. Significant pain relief was observed one‐week post‐injection, with rates nearing 100% in the following 47 weeks. Mean pain relief rate, presented as a percentage relative to baseline, was calculated. ***p < 0.001. Adapted with permission.^41^ Copyright © 2021 International Society for Cell & Gene Therapy. BMMSC, bone marrow mesenchymal stem cells; MRI, magnetic resonance imaging; MSCs, mesenchymal stem/stromal cells; UCB‐MSCs, umbilical cord blood‐derived mesenchymal stem cells.

Bone marrow mesenchymal stem cells (BMMSCs) are the predominant source of MSCs for the treatment of OA. Compared to adipose‐derived mesenchymal stem cells (ADSCs)^21^, ^22^ and umbilical cord blood‐derived mesenchymal stem cells (UCB‐MSCs),^23^ BMMSCs have a greater capacity for chondrogenic differentiation and are therefore currently the most widely used for OA treatment.^24^ Numerous preclinical studies have validated the effectiveness of BMMSCs in various animal OA models, including sheep,^25^ rats,^26^ mice,^27^ rabbits,^28^ horses,^29^ and guinea pigs.^30^ Similarly, BMMSCs alone have been shown to be effective in relieving knee pain and improving cartilage quality in OA patients in various clinical studies.^31^, ^32^, ^33^ In addition, treatment with BMMSCs at higher doses (50 × 10^6^ or 100 × 10^6^) significantly improved pain levels, knee mobility, and radiological findings in patients compared to low‐dose (10× 10^6^) stem cell therapy,^34^, ^35^ and more effectively protected cartilage and reduced synovial inflammation,^36^ results that pave the way for future phase III clinical trials. Although BMMSCs have achieved significant efficacy in animal experiments and clinical studies, their efficacy compared to UCB‐MSCs needs to be further evaluated. The results of animal experiments showed that BMMSCs alleviated OA progression in horses better than UCB‐MSCs, this is fully reflected in MRI^29^ (Figure 2a,b). In contrast, in clinical studies comparing bone marrow aspiration and concentration (BMAC) with allogeneic human UCB‐MSCs, one of the earliest sources of BMMSCs treatment, there was no significant difference observed in the treatment of medial knee unicondylar OA between the two groups. However, UCB‐MSCs were found to be more effective than BMAC in regenerating articular cartilage.^37^, ^38^ These results suggest that more clinical trials are needed to accurately assess the dose and efficacy of BMMSCs.

Although ADSCs do not have the same chondrogenic capacity as BMMSCs,^22^ they are becoming one of the hot spots of research in regenerative medicine because they are denser in tissue, proliferate faster in culture, are less likely to be affected by culture expansion and trigger senescence than BMMSCs,^37^ and can be obtained in a less invasive and cost‐effective manner through liposuction or arthroscopic surgery. In general, BMMSCs are the most common source of cells for OA treatment, followed by ADSCs.^38^ Various preclinical and clinical studies^39^, ^40^, ^41^, ^42^, ^43^ have shown that the use of ADSCs alone can effectively alleviate OA progression. Mei et al demonstrated that ADSC treatment can prevent cartilage degeneration in rat OA model^43^ (Figure 2c,d). And Chen et al. also found that IA injection of infrapatellar fat pad‐derived mesenchymal stromal cells (IFP‐MSC) appeared to be effective in ameliorating the symptoms of knee osteoarthritis^41^ (Figure 2e). Moreover, ADSCs isolated from different tissues (subpatellar fat, gluteal fat, and abdominal fat) were able to downregulate OA inflammatory factors and chemokines, suggesting that the anti‐inflammatory effects of ADSCs may not be dependent on the adipose tissue source or donor.^44^ By combining with chondrocytes, ADSCs could protect chondrocytes from oxidative stress, promote hyaline cartilage formation in rats, and inhibit chondrocyte apoptosis in *vivo*.^45^ Mixing with platelet‐rich plasma (PRP) improved patellofemoral OA joint function without complications, but no relevant changes were detected on magnetic resonance (MR) images.^46^ Compared to PRP alone, ADSCs with PRP, the effective doses of ADSCs for OA were also screened in clinical trials, which showed that doses of 5 × 10^7^ and 1 × 10^8^ ADSCs resulted in maximal improvement in knee function and pain.^47^, ^48^ Muthu's research^49^ indicates that MSCs derived from adipose tissue are superior to bone marrow in terms of safety, reliability, and effectiveness, with significant improvements in pain management and functional outcomes. Ehioghae et al. also believe that ADMSC has better efficacy in treating OA compared to other stem cell types, including human embryonic stem cells (hESC) and induced pluripotent stem cells (iPSC).^50^

Increasingly, MSCs from other tissue sources are being used in OA studies. It has been shown that synovial membrane‐derived MSCs (SMMSCs) have a higher chondrogenic potential than MSCs from the subpatellar fat pad, adipose tissue, and bone marrow.^51^ There have been reports on the treatment of OA with SMMSCs in preclinical^52^, ^53^, ^54^ and clinical studies.^55^ Xiao and colleagues demonstrated that transplanting SMSCs and inducing cartilage formation in place using E7‐Exosomes (E7‐EXO) to deliver Kartogenin (KGN) could be a progressive approach for stem cell therapy in OA.^56^ Umbilical cord‐derived mesenchymal stem cells (UC‐MSCs),^57^, ^58^, ^59^, ^60^ UCB‐MSCs,^61^, ^62^ Placental mesenchymal stem cells (PMSCs),^63^ and Neonatal mesenchymal stem cells (NMSCs),^64^ among other MSCs, are also increasingly studied in the treatment of OA.

### 2.2 Embryonic stem cells and induced pluripotent stem cells

MSCs have been increasingly explored as a therapeutic approach for OA, although in preclinical and clinical studies, their use is subject to many limitations, including donor heterogeneity and instability of cell quality.^65^, ^66^ In the last few years, the rapid development of pluripotent stem cell research has made them available as a new source for the derivation of MSCs. ESCs and iPSCs can be an unlimited source of differentiated cells for cell therapy.

**FIGURE 3:**
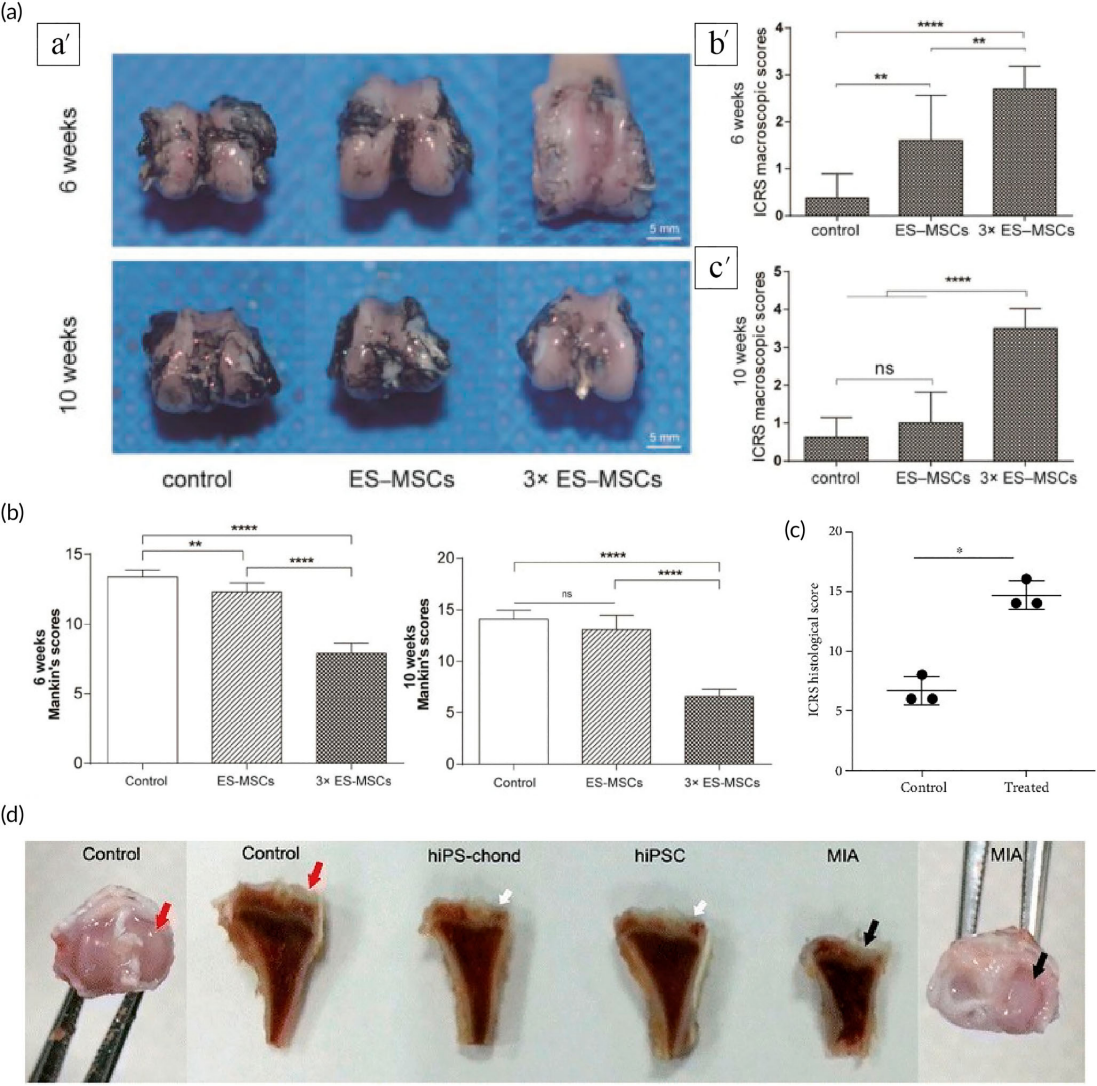
Therapeutic effects of ESCs and iPSCs on cartilage defect. (a) (a′) Macroscopic features of the femoral condyle, illustrated by India ink, were examined in three specimens per group at 6 and 10 weeks post‐initial injection. (b′) International Cartilage Repair Society (ICRS) macroscopic scores of the femoral condyle for all groups at 6 after the first injection. (c′) ICRS macroscopic scores of the femoral condyle for all groups at 10 weeks after the first injection. Error bars represent 95% confidence intervals (CI). ** *p* < 0.01, **** *p* < 0.0001, ns = no significance. (b) The corresponding modified Mankin scoresfor all groups. Error bars represent 95%CI. ** *p* < 0.01, **** *p* < 0.001, ns = no significance. Adapted with permission.^65^ Copyright © 2021 The Author(s). (c) The ICRS histological score evaluated tissue regeneration in rabbit articular cartilage defects 12 weeks after treatment. Scores in the iPSC‐MSC‐chondrocytes group were significantly higher than those in the control group (*n* = 3), *p = 0.02, analyzed using the Mann–Whitney *U* test. Adapted with permission.^75^ Copyright © 2020 The Author(s). (d) Macroscopic images of tibia at 15 weeks post injection. The control tibia showed no cartilage lesions in both the medial and lateral compartments of the tibial plateau (red arrow), while the monosodium iodoacetate (MIA)‐injected tibia exhibited severe cartilage lesions specifically on the medial tibial plateau (black arrow). Notably, observable repair was evident in the knee transplanted with iPS‐derived chondrocytes (white arrow). All the arrow point to medial tibial plateau. Adapted with permission.^77^ Copyright © 2016 The Author(s). ESCs, embryonic stem cells; iPSCs: induced pluripotent stem cells; MSCs, mesenchymal stem cells.

ESCs represent the inner cell mass of the blastocyst and have the capacity for pluripotent differentiation, which allows them to form three germ layers (ectoderm, endoderm, and mesoderm), giving rise to all cell types of the body. Therefore, ESCs are the best tool for tissue regeneration,^67^ and more than 30 clinical trials worldwide are currently evaluating the therapeutic efficacy of ESC‐derived cell products.^68^ In OA treatment, ESC‐derived MSC significantly improved the cartilage status of the femoral condyle in a rat OA model, with higher articular cartilage proteoglycan content and less cartilage loss^65^ (Figure 3a,b). Pluripotent stem cell‐derived MSCs (PSC‐MSCs) in combination with ESC‐MSCs enhanced the efficacy in rabbit OA.^69^ ESCs can also be injected into the joint cavity in the form of microspheres and reduced joint swelling, increased knee extensibility, slowed OA progression, and gradual recovery of damaged joint space, osteophytes, and exudative patellar ligaments were observed in rhesus monkeys suffering from OA.^70^ However, because ESCs are derived from embryos, their use has raised ethical concerns.

In 2006, iPSCs were first generated from mouse embryonic and adult fibroblasts by overexpression of four transcription factors, Sox2, Oct4, Klf4, and c‐Myc.^71^ Together, these four transcription factors reprogrammed somatic cells to regain pluripotency.

iPSCs share many characteristics with ESCs, including pluripotency, cell morphology, gene expression, and proliferative capacity. Furthermore, iPSCs are derived from a patient's own somatic cells, which helps circumvent the ethical controversies tied to the use of ESCs and reduces the risk of autoimmune responses, thus enhancing their suitability for patient‐specific therapies.^72^ However, it is crucial to consider the potential risks associated with iPSCs, such as the possibility of tumor formation.^73^ This consideration is essential for a balanced view of their utility in therapy and underscores that while iPSCs offer significant advantages, they may not be “ideal” in the absolute sense but rather “almost ideal.” However, due to the regulation of epigenetics and metabolic levels, the chondrogenic potential of iPSCs may be related to the disease specificity of donor cells.^74^ In OA therapy, iPSCs are first induced to differentiate into MSCs and subsequently into chondrocytes, and these iPSC‐MSC‐chondrocytes can effectively repair cartilage defects in rabbit OA models through anti‐inflammatory and anti‐catabolic mechanisms^75^ (Figure 3c). A single intra‐articular injection of iPSCs‐derived chondrocytes also effectively promoted cartilage repair in rats.^76^ iPSCs‐derived cartilage structures, with shapes and sizes suitable for repairing larger defects, may contribute to cartilage surface remodeling in articular cartilage defects.^72^ Zhu et al.'s research also suggested that hiPSC may be an effective and clinically translatable method for OA cartilage tissue regeneration^77^ (Figure 3d). Moreover, extracellular vesicles derived from iPSC have the potential for chondrocyte regeneration and OA therapy, which have been fully validated in rabbit models.^78^ However, iPSCs, like ESCs, carry a clinical risk of teratoma formation in *vivo*, and therefore their therapeutic applications remain mostly at the research stage，further research is needed to address the potential risks of tumorigenesis associated with hiPSCs.^73^

## 3 PRETREATMENT STRATEGIES OF STEM CELLS

The balance between the physiological and pathological state of the tissue severely affects the repair process. Most of the stem cells used in clinical applications come from adults or the elderly, and therefore the function of these cells may be impaired.^79^ OA joints have complex pathological changes with inflammation, hypoxia, and inadequate blood supply. In addition, clinical data suggest that the outcome of stem cell‐based therapies can be affected by drugs or infections.^80^ These factors may lead to disappointing results of stem cell therapy. To overcome these challenges, progressively some cell pretreatment tools have been developed. Stem cell pretreatment is a key strategy to improve stem cell function in *vitro* and in *vivo* and it will help to improve the efficacy of stem cells for transplantation in tissue engineering and regenerative medicine.^81^ A variety of pretreatment methods such as physical factors, drugs, cytokines, and hypoxic conditions can significantly enhance the biological functions of stem cells.^82^

### 3.1 Physical strategy

#### 3.1.1 Physical stimulation

**FIGURE 4:**
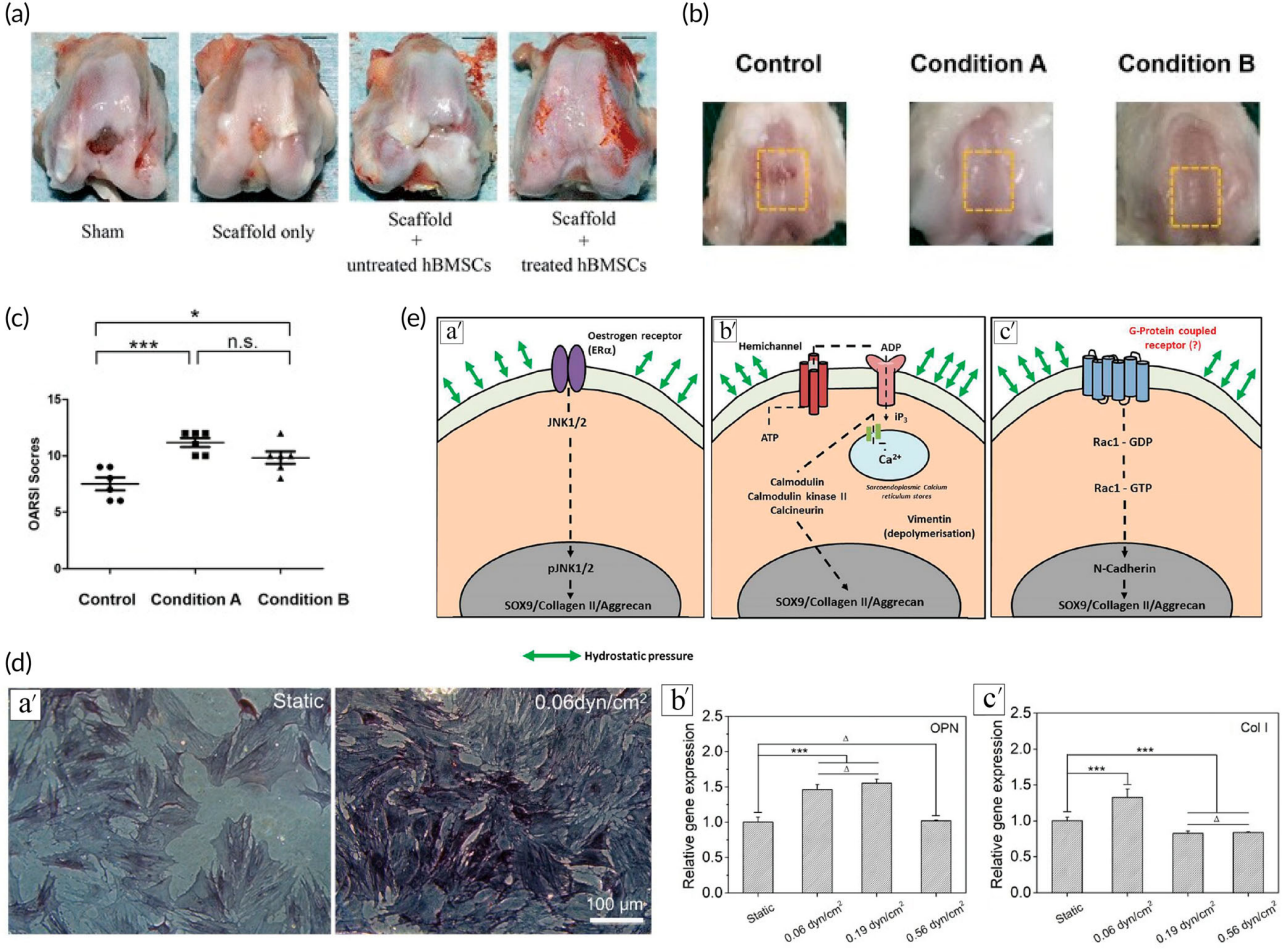
Physical strategies to improve cartilage differentiation of MSCs. (a) Evaluate the overall repair results based on the macroscopic appearance observed in each group of cartilage repair models. Sham: Sham surgery group; Scaffold only: Scaffold only group. Adapted with permission.^88^ Copyright © 2018 The Author(s). (b) Macroscopic view of nanosecond pulsed electric fields (nsPEFs)‐preconditioned MSCs enhanced cartilage regeneration in *vivo*. condition A: nsPEF preconditioning of 10 ns at 20 kV/cm; condition B: nsPEF preconditioning of 100 ns at 10 kV/cm. (c) ICRS Macroscopic score of joint. *n* = 6 per group. condition A: nsPEF preconditioning of 10 ns at 20 kV/cm; condition B: nsPEF preconditioning of 100 ns at 10 kV/cm. Adapted with permission.^89^ Copyright © 2019 The Author(s). (d) Osteogenesis of BMSCs under varied fluid shear stress (FSS) after 7 days in growth medium was studied. (a′) Phase‐contrast micrographs depicted cells under different FSS levels stained for alkaline phosphatase (ALP), an osteoblast indicator. (b′) and (c′) Statistical analysis showed relative gene expression (OPN, col I) in BMMSCs under different FSS conditions. “***”: *p* < 0.001, “Δ”: *p* > 0.05. BCIP/NBT alkaline phosphatase color development kit: A kit from Beyotime Biotechnology Co., Ltd. (China); OPN: An RT‐PCR primer sequences of the tested genes. Adapted with permission.^93^ Copyright © 2021 The Author(s). (e) Schematic diagrams summarize pathways influenced by hydrostatic pressure during MSC chondrogenesis. (a′) Pressure upregulates estrogen receptor, triggering the estrogen receptor pathway and activating anabolic responses via c‐Jun N‐terminal kinases (JNK). (b′) Hydrostatic loading activates voltage‐gated calcium ion channels and calcium stores (SERCs) through purinergic signaling, involving ATP release and interaction with purine receptors, stimulating calcium signaling. (c′) Pressure‐induced chondrogenesis affects cytoskeletal structure via GTPases, promoting the anabolic response by activating N‐cadherins, leading to MSC condensation and subsequent chondrogenesis. Adapted with permission.^96^ Copyright © 2019 The Author(s). BMMSCs, bone marrow mesenchymal stem cells; MSCs, mesenchymal stem cells; RT‐PCR: reverse transcription‐polymerase chain reaction.

In recent years, studies have shown that physical stimulation affects the differentiation of MSCs.^83^, ^84^ Based on OA treatment, the Low‐intensity pulsed ultrasound (LIPUS) can promote the cartilage formation of MSCs by inhibiting autophagy^85^ and also enhance the migration of rat BMMSCs by activating autophagy, improving its protective effect on rat OA cartilage.^86^ In addition, LIPUS antagonizes IL‐1β and promotes the expression of collagen type II (Col II) and Sox9 in MSCs.^87^ Radiation shock waves significantly promote the proliferation and self‐renewal of human BMMSCs and accelerate the cartilage repair process in rabbits^88^ (Figure 4a). Nanosecond pulsed electric fields (nsPEFs) pretreatment of porcine BMMSCs can activate the JNK/CREB‐STAT3 pathway to enhance the chondrogenic properties of BMMSCs and enhance the regeneration of cartilage defects in *vivo* in rats,^89^ and nsPEFs pretreatment could synergize with ghrelin to enhance the chondrogenesis of rat BMMSCs in *vitro* (Figure 4b,c). In addition, nsPEFs pretreatment can enhance cartilage formation in BMMSCs in *vitro* and cartilage regeneration in *vivo* in rats in concert with ghrelin.^90^ Sendera et al. concluded that electromagnetic fields have different effects on the proliferation of a variety of MSCs, possibly by increasing the number of cells that can differentiate and secrete cytokines and growth factors.^91^

#### 3.1.2 Mechanical stimulation

With the development of understanding of stem cell differentiation, the regulatory effects on directed differentiation of stem cells through mechanical forces have been more focused on, especially to chondrogenesis because articular motion is a combination of compressive, tensile, and shear deformations.^92^, ^93^, ^94^ Jing et al.^93^ found that BMSCs osteogenesis is highly sensitive to fluid shear stress (FSS) (Figure 4d). O Schätti^95^ et al used compression, shear, or a combination of both stimuli onto MSCs in fibrin/polyurethane composites to demonstrate that the application of shear and dynamic compression resulted in a significant increase in chondroblast gene expression, especially increased glycosaminoglycan (GAG) sulfate and type II collagen level. G Pattappa et al.^96^ found that enhancing the hydrostatic pressure has a positive influence on MSC chondrogenesis, increasing both the expression of genes and the level of matrix proteins such as SOX9, MMP13 and TGF (Figure 4e). In addition, a theory that cycle compression has a better effect on the chondrogenesis of MSCs was came up by Daniel Pelaez.^97^ We hold that pre‐treat MSCs by mechanical forces will mimic an external physiological environment similar to joints, which induced MSCs to transform into cartilage cells. However, the frequency, strength and combination of force stimulations, and the relationship between stimulations and types of cartilage remain unclear and need more studies to figure out.

### 3.2 Biological strategy

**FIGURE 5:**
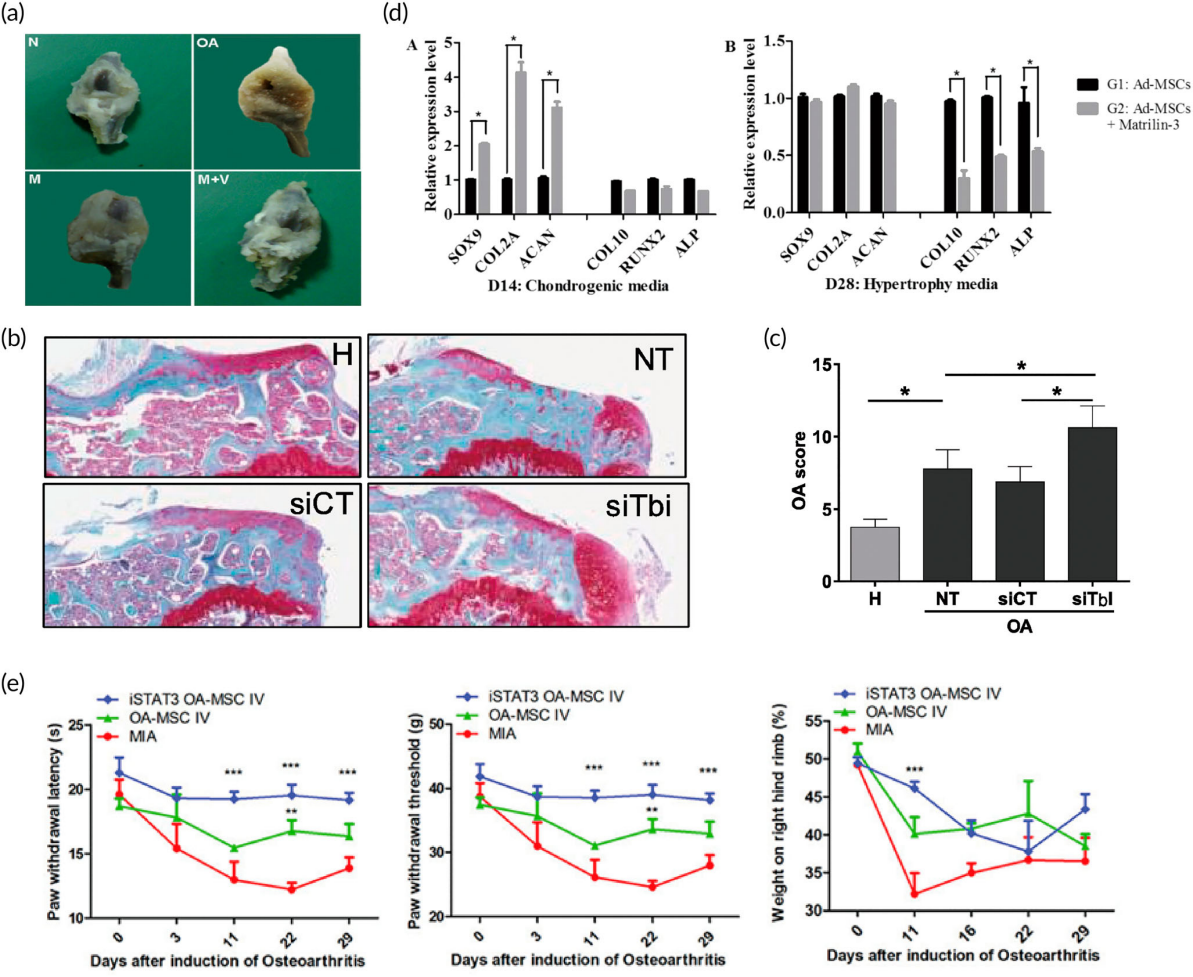
Biological strategies to improve cartilage differentiation of MSCs. (a) Morphological features of knee joints showed yellowish fibrocartilage in OA joints. Transplanting Vitamin E‐treated MSCs markedly reduced fibrosis. Adapted with permission.^98^ Copyright © 2016 Osteoarthritis Research Society International. (b) Impact of siTGFBI‐hMSCs in collagenase‐induced osteoarthritic (CIOA) mice. Histological images compared healthy (H) mice to CIOA mice untreated (NT) or treated with hMSCs transfected with control (siCT) or anti‐TGFBI (siTBI) mRNAs. (c) OA score of histological sections of knee joints of the CIOA mice. Results are expressed as the mean ± SEM; *: *p* < 0.05; ****: *p* < 0.0001. Adapted with permission.^99^ Copyright © 2019 Elsevier Ltd. (d) Matrilin‐3 inhibits Ad‐MSC‐induced chondrocyte hypertrophy. (A) mRNA expression of chondrogenic, hypertrophy, and ossification markers on day 14. G1: Control, G2: Ad‐MSC + matrilin‐3. *: *p* < 0.05, **: *p* < 0.01, ***: *p* < 0.001. Adapted with permission.^100^ Copyright © 2020 The Author(s). (e) Reduced OA severity observed with intra‐articular (IA) administration of STAT3 signaling inhibition (iSTAT3) OA MSCs in MIA‐induced OA rats. (a) Wistar rats underwent OA induction via IA injection of MIA. OA rats received IA injections of normal (Nor‐) MSCs, OA‐MSCs, or iSTAT3 OA‐MSCs. Pain behavior was assessed via mechanical hyperalgesia using a dynamic plantar esthesiometer and incapacitance meter, with paw‐withdrawal latency (PWL) and paw‐withdrawal threshold (PWT) quantified. Adapted with permission.^101^ Copyright © 2018 The Author(s). MIA, Monoiodoacetate; MSCs, mesenchymal stem cells; OA: osteoarthritis.

Many drugs or biokines have properties such as low solubility, instability, and rapid systemic elimination, resulting in poor bioavailability, so the pretreatment of stem cells with biokines to enhance the therapeutic effect of stem cells on OA is another pretreatment strategy. Vitamin E pretreatment enabled rat BMMSCs to resist hydrogen peroxide‐ (H_2_O_2_)‐induced oxidative stress and increased cartilage matrix proteoglycan content in *vivo* ^98^ (Figure 5a). TGF‐β‐induced gene product‐h3 (TGFBI/BIGH3) pretreatment improved the ability of human BMMSCs to treat OA in *vitro* and inhibited cartilage and bone degradation in *vivo*. The chondroprotective effect of MSCs is attributed to the presence of TGFBI mRNA and protein in EVs^99^ (Figure 5b–d). Matrilin‐3 prevents hypertrophy of human ADSCs‐derived chondrocytes, and injection of matrilin‐3 pretreated ADSCs pellets into the articular cavity of OA mice significantly improves cartilage regeneration and prevents subchondral osteosclerosis.^100^ Treatment of OA patient‐derived ADSCs with the STAT3 inhibitor STA21 significantly enhanced the anti‐inflammatory function and migratory potential of ADSCs and slowed the progression of OA in rats^101^ (Figure 5e). Rizzo et al. have shown that the use of MSC or MSC derived products alone or in combination with hemolytics to control patient symptoms and potentially alleviate OA progression has encouraging potential.^102^

### 3.3 Hypoxia strategy

**FIGURE 6:**
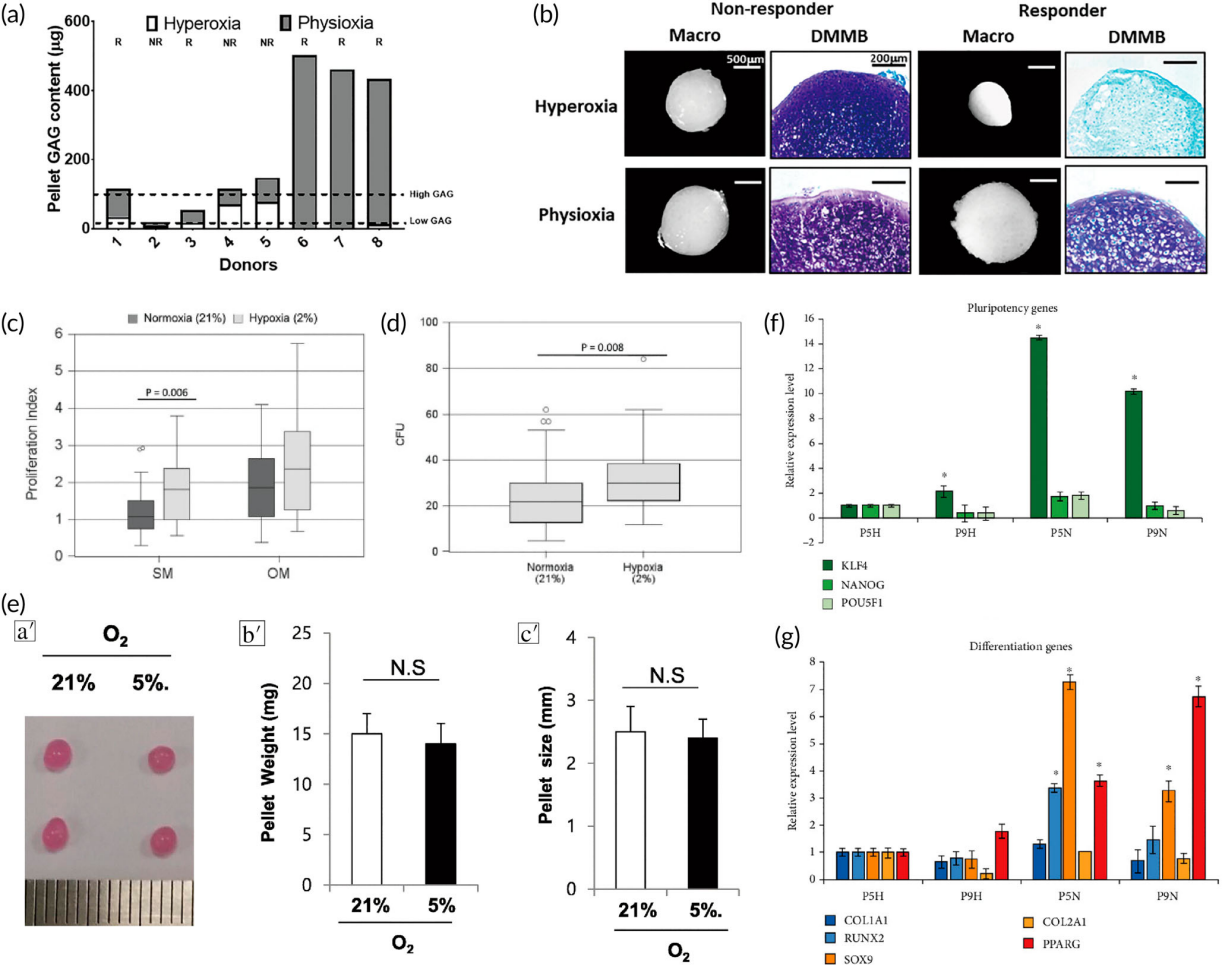
Biological strategies to improve cartilage differentiation of MSCs. (a) Pellet GAG content under physioxia and hyperoxia conditions for chondrogenic pellets. Dotted lines indicate thresholds for high (physioxia non‐responsive) and low (physioxia responsive) GAG donors. *R* represents physioxia responders, and NR indicates physioxia non‐responders. (b) Representative macroscopic and DMMB‐stained chondrogenic pellets of physioxia non‐responders and responders. Data are presented as mean ± standard deviation (SD); *n* = 5. * *p* < 0.05. DMMB dye: 18 μg/mL in 0.5% ethanol, 0.2% formic acid, 30 mM sodium formate, ph = 3. Adapted with permission.^103^ Copyright © 2020 The Author(s). (c) In *vitro* cell growth of BMMSCs maintained for 6 days under normal or hypoxic conditions. OM refers to osteogenic medium, and SM indicates standard medium. (d) Clonogenicity of BM‐MSCs (number of colony‐forming units [CFU] following BM‐MSC culture with standard medium) under either normoxic or hypoxic conditions. Adapted with permission.^104^ Copyright © 2016 International Society for Cellular Therapy. (e) SDSCs were pelletized and subjected to chondrogenic differentiation under normoxic and hypoxic conditions for 21 days. The effects were assessed by examining pellet morphology, size, and weight. Adapted with permission.^110^ Copyright © 2018 The Author(s). (f) Gene expression levels were measured via qRT‐PCR for cells cultured under hypoxic and normoxic conditions. (a′) Pluripotency genes KLF4, NANOG, and POU5F1 were analyzed. (b′) MSC differentiation genes, including osteogenic markers COL1A1 and RUNX2, chondrogenic markers SOX9 and COL2A1, and adipogenic marker PPARG, were assessed. Expression levels were normalized to GAPDH and P5H, with * indicating significant differences (*p* < 0.05) compared to the P5H sample. Adapted with permission.^112^ Copyright © 2020 The Author(s). BMMSCs, bone marrow mesenchymal stem cells; GAG, glycosaminoglycan; MSCs, mesenchymal stem cells; SDSCs, synovium‐derived stem cells.

It has been shown that in a rabbit knee trauma and focal early OA model, treatment of MSCs cultured under physiological oxygen conditions significantly improved cartilage repair scores compared to MSCs cultured under hyperoxic conditions^103^ (Figure 6a,b). Compared to normoxic conditioned BMMSCs under hypoxic conditions had a greater capacity for proliferation and colony formation compared to BMMSCs under normoxic conditions^104^ (Figure 6c,d), and hypoxic pretreatment combined with microbubble‐mediated ultrasound can significantly promote the migration ability of human BMMSCs.^105^ Since articular cartilage tissue is under hypoxic conditions in *vivo*,^106^ researchers have explored hypoxic conditions as a strategy to enhance cartilage differentiation of MSCs to better mimic the natural articular cartilage environment,^107^, ^108^, ^109^ but different findings have emerged. Bae et al. found that SDSCs under hypoxic conditions promote chondrogenesis by promoting the synthesis of ECM matrix^110^ (Figure 6e). Hypoxic culture conditions do not increase cartilage formation in equine SMMSCs or BMMSCs but may downregulate the expression of COL10A1, a marker of hypertrophy in SMMSCs.^111^ Pluripotency, osteogenesis, chondrogenesis, and lipogenesis genes were expressed at higher levels in ADSCs under normoxic culture compared to hypoxia, and the expression levels decreased with an increasing number of passages^112^ (Figure 6f). Zhang et al. found that hypoxia cultured ADSC secreted exosomes (hypoxia ADSC Exos) carry effective miRNA, which can not only improve the function of normal human articular chondrocytes (HAC) but also alleviate HAC inflammation and OA progression.^113^ Therefore, more studies are needed to confirm the role and mechanism of hypoxic pretreatment in the treatment of OA by stem cells.

## 4 COMBINED WITH BIOMATERIALS

Stem cells can be effective in alleviating the progression of OA. However, the low retention and survival of stem cells at the injection site cause the requirements of high doses of cells or repeated injections, which is not economically feasible and creates additional complication risks. To address this issue, a suspension environment is needed to allow stem cells to coalesce, as intercellular communication is crucial for cell survival.^114^ Appropriate biomaterials can not only increase the half‐life of stem cells after injection but can also enhance the chondrogenic differentiation of stem cells,^115^ which can effectively enhance the therapeutic effect of stem cells for OA.

Injecting MSCs into the joint cavity of OA rats with microcarriers can reduce the dose of MSCs without reducing the repair effect.^116^, ^117^ Encapsulation of human BMMSCs with sodium alginate microspheres was able to modulate the paracrine response of BMMSCs in the simulated OA microenvironment, prolonging the residence time of MSCs in the joint cavity,^118^ enhancing the therapeutic effect of MSCs on OA, reducing further cartilage degeneration and subchondral osteosclerosis,^119^ but no significant improvement in pain, synovial inflammation, and cartilage damage.^120^ Johnbosco and colleagues observed a significant material‐dependent effect when injecting both unencapsulated and microencapsulated MSCs into the joints of OA rats, resulting in reduced cartilage degradation and matrix loss.^121^ Simultaneous encapsulation of transforming growth factor‐β3 (TGF‐β3) and MSCs in PLGA‐P188‐PLGA microspheres not only optimizes the release of TGF‐β3 in the joint cavity of OA mice but also facilitates the in‐situ differentiation of MSCs and formation of new cartilage‐like tissues.^122^ Self‐assembled peptide hydrogels can also be used for the encapsulation of MSCs and effectively prevent apoptosis of articular chondrocytes in OA rats, altering subchondral bone mineral density and increasing GAG synthesis, effectively slowing the progression of cartilage destruction in OA.^123^, ^124^ Similarly, piggybacking on a collagen injectable scaffold improved the chondrogenic effect of human ADSCs and cartilage regeneration in rabbit OA models.^125^ The combination of UCB‐MSCs promoted proteoglycan and type II collagen synthesis in the rabbit OA model and enhanced anti‐inflammatory activity in rabbit joints and synovial fluid.^126^ Furthermore, Yang and colleagues demonstrated that incorporating essential cell components, such as chondrocytes and mesenchymal stem cells, into bioprinted structures plays a crucial role in promoting tissue regeneration and repair.^127^ In clinical applications, arthroscopically guided administration of fibrin glue mixed with (Greenplast kit W, Greencross) and human ADSCs implanted at the lesion site under arthroscopic guidance significantly improved clinical outcomes, with a significant increase in the number of patients treated at 5 and 7 years after treatment. The patients' survival rates of with knee OA at 5, 7, and 9 years after treatment were 99.8%, 94.5%, and 74.5%, respectively.^128^ But with respect to injected sources of MSCs, the most appropriate cell sources for treating OA have been unclear yet. The summary is shown in Table 1.

**TABLE 1:** Combined with biomaterials of stem cells in OA.

Biomaterial	Stem cell	Model(s) used	Experimental subject	Administration and dose	Function	Reference
Gelatin microcarriers	HUC‐MSCs	Bilateral knee OA was induced by anterior cruciate ligament (ACL) transection.	Rat	1 × 10^5^ hUC‐MSCs in about 0.5 to 0.7 mg microcrystals in saline with a total volume of 100 mL were injected at 4 weeks after ACL surgery.	The use dose of MSCs can be reduced without reducing the repair effect.	116, 117
Sodium alginate microspheres	hBMMSCs	Blunt dissection to expose the medial collateral ligament (MCL), a full‐thickness cut was made through the meniscus at its narrowest point.	Rat	1 d post‐surgery, the cell dose (5 × 10^5^ cells/knee) used for injection was the maximum concentration that could be encapsulated and delivered in a 50 μL volume.	Early manifestations of protective effects on joint cartilage reduced joint cartilage swelling and increased surface roughness.	118
	hBMMSCs	A surgical instability animal model, medial meniscal tear (MMT), was used to induce OA.	Rat	5 × 10^5^ encapsulated hMSC in HBSS should be injected within 2 h (cells were stored at 4°C until injection) after 1 week of OA induction.	Reduced further cartilage degeneration and subchondral sclerosis.	119
	BMMSCs	Monoiodoacetate‐induced osteoarthritis.	Rat	Inject 0.8 × 10^5^ ± 0.1 × 10^5^ hMSC encapsulated in alginate beads in both knees after 1 week of OA induction.	Regulation of paracrine response and prolongation of MSCs in the joint cavity and enhanced treatment of OA by MSCs.	120
PLGA‐P168‐PLGA and TGF‐β3	hBMMSCs	Collagenase‐induced osteoarthritis (CIOA) model.	Mice	2.5 × 10^5^ cells were added to 0.5 mg PAM or PAM‐T in 1 mL of adhesion medium. After 4 h, eliminate serum and resuspend in a solution of different concentrations of CMC; mice received MSCs pre‐incubated with PAM or PAM‐T (7 mL total).	Optimize the release of the TGF‐β3 in the articular cavity of OA mice and is conducive to the in situ differentiation of MSCs and the formation of new cartilage‐like tissues.	122
Self‐assembled peptide (SAP) hydrogel	BMMSCs	Cut the anterior cruciate ligament and medial collateral ligament, and completely remove the medial meniscus.	Mice	1×10^6^ cells/mL of rBMSCs were mixed with the same volume of 1% peptide gel, and 0.2 mL of each compound was injected into the articular cavity 3 weeks after the induction of OA surgery.	Effective prevention of apoptosis of articular chondrocytes in OA rats changes the density of subchondral bone minerals and increases GAG synthesis.	123
Carrying collagen injectable scaffold	ADSCs	Methylated bovine serum albumin antigen‐induced rabbit knee joints evolving into OA.	Rabbit	Knee joints of the right hind paw of the rabbits of the experimental groups were injected with 1 × 10^6^ MSCs, 0.5 mL of MCH separately or with 1 × 10^6^ MSCs, 0.5 mL of the growth culture medium without serum with 5 mg DPC separately.	Improved effect of human ADSCs on cartilage regeneration of rabbit OA model.	125
ECM Materials	hUCB‐MSCs	Cutting medial parenchyma of the ACL and the meniscus ligament with a surgical blade.	Rabbit	UCB‐MSCs (2.5 × 10^6^ cells) and/or CAM (20 mg/mL) in each group were suspended in 200 μL of saline and transplanted intra‐articularly at 8 weeks after the ACLT surgery.	The synthesis of proteoglycan and type II collagen in the rabbit OA model was promoted, and the anti‐inflammatory effect in rabbit joints and synovial fluid was enhanced.	126
Fibrin glue	ADSCs	Full‐thickness cartilage lesion, Kellgren‐Lawrence26 OA grade 1 or 2.	Human	Arthroscopic implantation contained an average of 8.11 × 10^6^ stem cells.	Significantly improve clinical results.	128

Abbreviations: ACLT, anterior cruciate ligament transection; ADSCs, adipose‐derived mesenchymal stem cells; BMMSCs, bone marrow mesenchymal stem cells; hBMSC, human bone marrow‐derived stem cells; MSCs: mesenchymal stem cells; OA, osteoarthritis; UCB‐MSCs, umbilical cord blood‐derived mesenchymal stem cells.

## 5 STEM CELL ENGINEERING

To overcome the problem of short survival time (1–2 weeks) of stem cells after injection,^129^, ^130^ in addition to the combination with biomaterials the low affinity of stem cells for the target tissue after injection can be addressed by cell engineering. (Table 2) DeJulius et al.^141^ summarized emerging RNA‐based technologies, including small interfering RNAs for gene silencing, microRNA and anti‐microrNA for polygene regulation, mRNA for gene supplementation, and RNA‐guided gene editing platforms such as CRISPR‐Cas9, which may be used to treat OA therapy. Moreover, cell engineering strategies were further explored to incorporate into the framework of OA therapy by enhancing the disease‐dependent and spatio‐temporal regulation of therapeutic molecules. To make the cells target cartilage tissue, palmitic acid protein G (PPG) can be embedded in the cell membrane of human BMMSCs and then conjugated with type II collagen antibodies. The engineered stem cells were able to attach to the osteochondral surface within 48 h.^138^ In addition, the functional peptides on the cell membranes of BMMSCs can also be modified to take advantage of the fact that joint‐damaged chondrocyte glutaminase 2 (Transglutaminase 2, TGase2) to target chondrocytes for better therapeutic results.^131^

**TABLE 2:** Engineering application of stem cells in treating osteoarthritis in *vivo* and *vitro*.

Treated in *vivo*					
Cellular engineering substances	Targeting cells	Model(s) used	Administration and dose	Function	Reference
TGase2	Human BMMSCs	Use the scalpel to create PCDs in the rabbit's left and right femoral pulleys. Remove the cartilage to expose the deep surface.	TP‐modified MSCs (tMSCs; 4 × 10^6^ cells) prepared in *vitro* were injected intra‐articularly 4 weeks after establishing the injury model. Samples were retrieved for analysis 8 weeks after intra‐articular injection.	Target chondrocytes for better therapeutic results.	131
mRNA	Mice ADSCs	The medial meniscus was destabilized by cutting the medial meniscotibial ligament.	2 × 10^5^ADSCs, GFP‐ADSCs or IGF‐1‐ADSCs were stained with DiI and respectively injected into the knee joints of mice.	IGF‐1‐ADSCs had a superior therapeutic effect over native ADSCs demonstrated by lower histological OARSI score and decreased loss of cartilage ECM.	132
TNF‐α blocker Atsttrin gene	Mice C3H0T1/2	Anterior cruciate ligament transection.	Intra‐articular injections of MSC‐Atsttrin or MSC‐GFP (1 × 10^5^cells in 10 mL DMEM) were carried out at 1‐week post‐OA surgery.	Atsttrin enhanced the protective effect of MSCs and more effectively delayed the onset of OA.	133
IL‐4 gene	Mice ADSCs	Intra‐articular injection of monoiodoacetate (MIA) has been used to induce OA.	5 × 10^6^ cells were injected into the joint cavity, and the observation period after OA was 8 weeks.	IL‐4 MSC spheroids show better cartilage protection and pain relief than native MSCs.	134
Collagen type II (COL2) receptor integrin α10β	Horse ADSCs	Under arthroscopic guidance, horse talocrural model of impact‐induced articular injury.	20 × 10^6^ viable MSCs in 3 mL of phosphate‐buffered saline (PBS) were injected into the randomly assigned left or right joint.	Resulting in less cartilage damage, subchondral osteosclerosis and long‐term protection of the joint.	135
PDGF/HO‐1	Dog ADSCs	The right CrCL was transected via medial parapatellar arthrotomy.	1 mL canine HO‐1‐overexpressing MSCs (HO‐1‐MSCs)/PDGF‐overexpressing MSCs (PDGF‐MSCs) at the density of 2 × 10^7^ cells/mL.	Upregulate anti‐fibrotic and pro‐chondrogenic factors and exert immunomodulatory effects on OA chondrocytes and synovial cells.	136
PUMILIO1 protein	Mice MSCs	Induce OA by dissecting the medial meniscus‐ligament to destabilize the medial meniscus.	MSCs were replated at a density of 5000 cells/cm^2^ and were subcultured at 80% confluency. The lentivirus encoding PUM1 was injected one day after DMM surgery.	Protect MSCs from H_2_O_2_‐induced cellular senescence.	137

Treated in *vitro*				
Cellular engineering substances	Targeting cells	Administration and dose	Function	Reference
PPG	Osteochondral implant (OC implant)	Different concentrations of palmitic protein G (PPG) were added to 2 × 10^6^/mL stem cells, incubated 100 μ g/mL of the targeted antibody in PBS at 4°C for 1 h.	Demonstrated the preservation of the MSCs' ability to proliferate and differentiate chondrogenically in culture.	138
TGase2	Pig chondrocyte	Incubated an equal number (1 × 10^5^) of MSCs (wide‐type MSCs or tMSCs) and ACs (healthy ACs and ACs injured with ATRA treatment) for 30 min suspension culture in 1.5 mL tubes.	MSCs improved the function of injured ACs and enhanced the chondrogenic differentiation potential of MSCs.	131
mRNA	Mouse chondrocyte	Plated IGF‐1‐ADSCs onto the membrane of transwells, and chondrocytes were plated onto the bottom of 24‐well plates. Human interleukin 1 beta (IL‐1β) was added to the medium (10 ng/mL).	IGF‐1‐ADSCs increased anabolic markers expression of chondrocytes in the inflammation environment compared to untreated ADSCs.	132
TNF‐α blocker Atsttrin gene	Human chondrocyte	Seeded MSC‐Atsttrin onto the membrane of suitable Transwells. TNF‐α was added to the medium at a final concentration of 10 ng/mL when the cells grew to 70% confluence.	MSC‐Atsttrin plays a chondroprotective role to delay the progression of OA in *vitro* and enhancing the production of beneficial cartilage extracellular matrix.	133
IL‐4 gene	Rat chondrocytes	Approximately 5 × 10^5^ MSCs were incubated in 1 mL of medium for 12 h, and the secreted IL‐4 protein concentration was about 7 ng/mL.	IL‐4 MSC spheroids showed more substantial chondroprotective and anti‐inflammatory effects in an OA chondrocyte model in *vitro*.	134
IL‐1Ra gene	Horse chondrocyte	Chondrocyte spheroids were co‐cultivated with MSC/IL‐RA cells for an additional 24, 48, or 72 h.	Co‐cultivation of MSC/IL‐1Ra cells with osteoarthritic spheroids alleviates the severity of the osteoarthritic changes.	139
PDGF/HO‐1	Canine chondrocytes or synovial cells	The PDGF‐MSCs/ HO‐1‐MSCs (5.0 × 10^4^)—cocultured cells were incubated further for 24 or 72 h.	The co‐cultured MSCs highly expressed genes that contributed to the maintenance of joint homeostasis.	136
miR‐410	Human chondrocyte	The miR‐410 or miR‐410‐in were induced to chondrocytes for 3–5 days， all cells were treated with 20 μL MTT at 5 mg/mL for 4 h.	Promote chondrogenic differentiation of human BMMSCs.	140
PUMILIO1 protein	Mouse chondrocyte	The genetically modified MSCs with constant PUM1 expression may be effective for the regeneration of damaged cartilage using in *vitro* cell culture system.	The genetically modified MSCs with constant PUM1 expression may be effective for the regeneration of damaged cartilage using in *vitro* cell culture system.	137

Abbreviations: ADSCs, adipose‐derived mesenchymal stem cells; BMMSCs, bone marrow mesenchymal stem cells; MSCs, mesenchymal stem cells; OA, Osteoarthritis; PCD, partial‐thickness cartilage defects; PDGF, platelet‐derived growth factor; tMSCs, TP‐modified MSCs.

Genetic modification has been used to optimize the treatment of stem cells and can significantly enhance the therapeutic effect of stem cells for OA. Chemically modified mRNAs can be transfected to obtain engineered cells with the ability to secrete insulin growth factor‐1 (IGF‐1). IGF‐1‐ADSCs can increase the expression of anabolic markers in inflammatory chondrocytes and effectively delay OA progression in mice.^132^ Transfection of tumor necrosis factor α (TNF‐α) blocker atsttrin gene into mouse mesenchymal stem cell line C3H10T1/2 cells engineered to suppress catabolic factor expression, promote cartilage extracellular matrix production, and delay OA progression in *vitro* and in *vivo*.^133^ Interleukin‐4 (IL‐4) gene transfected into rat ADSCs enhanced the anti‐inflammatory properties and chondroprotective effects of MSCs.^134^ Transfection of the Interleukin‐1 receptor antagonist (IL‐1Ra) gene into equine BMMSCs reduced the exacerbation of OA.^139^ High expression of COL2 receptors and integrin α10β on equine ADSCs used to treat equine OA models resulted in less cartilage damage and subchondral osteosclerosis and it may also protect joint cartilage from continuous degradation. And it is necessary to conduct long‐term research.^142^ Transfection of platelet‐derived growth factor (PDGF), hemoglobin factor or heme oxygenase‐1 (HO‐1) to obtain engineered ADSCs, which could upregulate anti‐fibrotic and pro‐chondrogenic factors and exert immunomodulatory effects on OA chondrocytes and synovial cells.^136^ Transfection of miR‐410 can promote chondrogenic differentiation of human BMMSCs through the Wnt signaling pathway, providing an opportunity to combine miRNA technology with MSCs therapy. The combination of miRNA technology and MSCs therapy for OA provides a proof‐of‐concept and experimental approach.^140^ Various preclinical and clinical advancements have been made in RNA chemical modification and RNA based drug delivery, showing promising prospects in preclinical models of OA, including the use of small interfering RNAs, microRNAs, anti microRNAs, messenger RNA, and CRISPR‐Cas9.^141^ Overexpression of PUMILIO1 can protect MSCs from H_2_O_2_‐induced cellular senescence even after lipopolysaccharide (LPS) or interleukin 1β (IL‐1β), PUMILIO1 overexpression enhances the chondrogenic potential of MSCs' chondrogenic potential.^137^ The summary is shown in Table 2.

## 6 STEM CELL THERAPY IN ANIMAL MODEL AND CLINICAL TRIAL

Due to ethic issues and special sources, prognosis and safe issues, application of MSCs requires more pre‐clinical data. Different animal models are used to improve their mechanisms, functions, and safety. As mentioned earlier, each animal has its own advantages and disadvantages in experimental evaluation, including sheep^25^ (Similar anatomy to human/arthroscopy assessment but various in cartilage thickness and dense subchondral bone), rats^26^ (Economic/easy handle but the joint is small), mice^27^ (Economic/easy handle but the joint is small), rabbits^28^ (Reach early skeletal maturity/simple husbandry but there is intrinsic healing ability and different weight‐bearing settings), horses^29^ (Largest model/chronic cartilage defects but the loading environment is hard to get), and guinea pigs^30^ (Large cartilage thickness defects but there are housing, size, and handling difficulties).^143^

Data from clinical trials is also important. BMMSCs have a greater capacity for chondrogenic differentiation and are therefore currently the most widely used for OA treatment, but their usage needs review by the ethics committee. Whether MSCs therapy is significant also needs clinical trials to demonstrate. We summarize some clinical studies in Table 3 on this field to have a comprehensive view.

**TABLE 3:** Summary of clinical studies on MSCs therapy.

Stem cells	Clinical trial status	Outcomes	Sample sizes	Trial registration	Reference
hUCB‐MSCs	A phase I/II—7 years of extended follow‐up, open‐label, single‐arm, single‐center	Allogeneic hUCB‐MSC product (Cartistem) is safe and improves the prognosis of OA.	Eight patients	‐	129
BM‐MSCs	A phase II, multicenter, randomized clinical trial	Treatment with BM‐MSC associated with PRGF® was a viable therapeutic option for osteoarthritis of the knee, with clinical improvement at the end of follow‐up.	60 patients	NCT02365142	14
haMPCs	A prospective, randomized, double‐blind, active‐controlled, phase IIb clinical trial	Significant improvements in joint function, pain, quality of life, and cartilage regeneration were observed in Re‐Join®‐treated knee OA patients with good tolerance in a period of 12 months.	53 patients	NCT02162693	142
UC‐MSCs	A controlled randomized phase I/II clinical trial	Repeated MSC dosing is superior to a single MSC dose and to hyaluronic acid.	29 patients	NCT02580695	60
MSCs (40 × 10^6^ cells)	A phase 1/2 single‐center, triple‐blind, randomized controlled trial with a placebo control.	Intra‐articular implantation of MSCs provided significant and clinically relevant pain relief over 6 months versus placebo and could be considered a promising novel treatment for knee OA.	43 patients	‐	33

Abbreviations: BMMSCs, bone marrow mesenchymal stem cells; MSCs, mesenchymal stem cells; OA, Osteoarthritis; UC‐MSCs, umbilical cord‐derived mesenchymal stem cells.

## 7 OUTLOOK AND CONCLUSION

In OA treatment in recent decades, stem cells showed unique advantages, including increasing cartilage formation, promoting cartilage cell proliferation and apoptosis, adjusting the synthesis and decomposition of the cartilage extracellular matrix metabolism, regulating immune response, inhibiting inflammation and paracrine effect, preclinical and clinical research has a wide range of applications.

With the development of stem cells therapy, more and more studies focus on certain clinical impacts of the direct transplantation of stem cells into target tissues such as tumorigenicity, immune incompatibility, and chromosomal aberrations. Meanwhile, the regenerative, anti‐inflammatory, immune regulation, and other functions of stem cells are primarily mediated through their paracrine activity. This insight has provided a new avenue for exploring MSC therapy using advanced pretreatment strategies. In this framework, exosomes serve as vehicles for delivering genes and drugs, thus facilitating targeted therapeutic interventions without implying that they act as carriers for cells themselves. Therefore, more observations and explorations are needed on these OA models, summarizing what types of cytokines can be secreted by various types of stem cells through paracrine effects, and exploring their signaling pathways to explain their deeper mechanisms of action，which provides a theoretical basis for further understanding the paracrine mechanisms of stem cell therapy. Reliable clinical data require rich clinical trials to demonstrate the safer and more effective clinical effects of stem cells. Second, a series of validation experiments on subject samples, such as RNA‐sequencing and proteomics sequencing, are needed to demonstrate the effectiveness of stem cell therapy. And studies have also shown that cell pretreatment strategies or engineered MSCs and their application in combination with tissue engineering have also yielded encouraging results, and their safety and effectiveness have been proven in various animal experiments. The future research direction may shift to clinical trials of tissue engineering, biomaterials, and stem cells for critically ill patients with clear intentions, which will provide new strategies for OA treatment.

## AUTHOR CONTRIBUTIONS

**Jianjing Lin:** Conceptualization; data curation; formal analysis; funding acquisition; investigation; methodology; project administration; resources; software; supervision; writing – original draft. **Jingtao Huang:** Conceptualization; data curation; formal analysis; investigation; validation; visualization; writing – original draft. **Zilu Jiao:** Conceptualization; investigation; validation; visualization; writing – original draft. **Mengyuan Nian:** Methodology; project administration; software; writing – original draft. **Canfeng Li:** Writing – original draft; writing – review and editing. **Yali Dai:** Conceptualization; investigation; writing – original draft; writing – review and editing. **Shicheng Jia:** Conceptualization; data curation; formal analysis; investigation; methodology; project administration; visualization; writing – original draft; writing – review and editing. **Xintao Zhang:** Funding acquisition; resources; software; supervision; writing – original draft; writing – review and editing.

## FUNDING INFORMATION

This study was supported by the National Natural Science Foundation of China (No. 82272568), Shenzhen “San‐Ming” Project of Medicine (No. SZSM202211019), Guangdong Basic and Applied Basic Research Foundation (2023A1515220019, 2022A1515220056) and “Merro” Young Physician Innovation and Development Project (GSKQNJJ‐2023‐004).

## CONFLICT OF INTEREST STATEMENT

The authors declare that they have no competing interests.

### PEER REVIEW

The peer review history for this article is available at https://www.webofscience.com/api/gateway/wos/peer-review/10.1002/btm2.10701.

## Supporting information


**Data S1:** Supporting Information.

## Data Availability

Data sharing is not applicable to this article as no new data were created or analyzed in this study.
