# Supplementary material for: Mesenchymal stem cells for osteoarthritis: Recent advances in related cell therapy
Source: Bioeng Transl Med. 2024 Aug 5;10(1):e10701. doi: 10.1002/btm2.10701 (PMC11711223; doi:10.1002/btm2.10701)
Supplement: Supplementary file 1 — Data S1: Supporting Information. [file BTM2-10-e10701-s001.zip › Additional file.docx]

**Additional File**

The additional files contained the RightsLink Printable License for several reused images within the document, specifically Figure 2E, Figure 5A, Figure 5B-C, and Figure 6C-D. The remaining reused other images, namely those in Figures 2 through 6, were covered under the CC BY license and have been appropriately standardized and cited as required. We thanked the authors and copyright holders of these materials for their figures and contributions.
